# Impaired and restored CD8^+^ T cell responses to a sequentially mutated spike epitope in Omicron variants

**DOI:** 10.3389/fimmu.2025.1684193

**Published:** 2025-11-06

**Authors:** Eun Joo Chung, Soyoung Park, Su-Hwan Kim, Ju-yeon Choi, Hye-Sook Jeong, Jae-Hoon Ko, Joon Young Song, Young Jae Lee

**Affiliations:** 1 Division of Clinical Research for Vaccine, Center for Vaccine Research, National Institute of Infectious Diseases, National Institute of Health, Korea Disease Control and Prevention Agency, Cheongju, Republic of Korea; 2 Division of Infectious Diseases, Department of Medicine, Samsung Medical Center, Sungkyunkwan University School of Medicine, Seoul, Republic of Korea; 3 Division of Infectious Diseases, Department of Internal Medicine, Korea University Guro Hospital, Korea University College of Medicine, Seoul, Republic of Korea

**Keywords:** SARS-CoV-2, COVID-19 vaccine, breakthrough infection, T cell epitope, Omicron variant, escape mutation

## Abstract

In the post-pandemic era, SARS-CoV-2 variants continue to circulate and evolve. T cell-mediated immunity is essential for antiviral defense, but its evasion by emerging variants remains poorly defined. In this study, we screened spike-derived CD8^+^ T cell epitopes using bioinformatic algorithms and validated them experimentally in COVID-19 vaccine recipients. LYNSASFSTF (LYN), located in the receptor-binding domain (S_368–377_), was identified as an HLA-A*24:02-restricted epitope harboring mutations observed in Omicron BA.1 (S371L, S373P, and S375F) and BA.2 (S371F, S373P, S375F, and T376A). BA.2 mutations more profoundly attenuated LYN-specific cellular immunity than those from BA.1 with T376A as a major contributor to immune evasion, as supported by structural analysis of altered peptide–HLA interactions. This effect was mitigated by BA.1/BA.2 breakthrough infection and appears unlikely to persist in the currently dominant NB.1.8.1 lineage, where the LYN sequence remains conserved. Our findings suggest that LYN may function both as an immune-evasive hotspot and as a cross-reactive epitope, underscoring the importance of continued epitope-level surveillance as future variants emerge.

## 1 Introduction

On May 5, 2023, the World Health Organization (WHO) officially declared the end of the global health emergency for coronavirus disease (COVID-19) (1). Nevertheless, the disease continues to threaten public health security as it transitions into an endemic phase (2, 3). Severe acute respiratory syndrome coronavirus 2 (SARS-CoV-2), the causative agent of COVID-19, continues to circulate globally, and is constantly evolving to evade both natural- and vaccine-induced immunity (4). Consequently, COVID-19 vaccines have been repeatedly reformulated to address newly emerging variants; however, the evolution of SARS-CoV-2 strains threatens to compromise immune protection (5–10), creating an ongoing cycle.

The Omicron variant (B.1.1.529), first reported in November 2021 (11), has remained the dominant backbone of global SARS−CoV−2 circulation through successive descendant lineages. After the initial BA.1 and BA.2 waves in early 2022, the XBB recombinant clade and its offshoot KP.3.1.1 predominated throughout 2023. In mid−March 2025, the JN.1−derived subvariant LP.8.1 accounted for the largest share of global sequences (12); however, since late April, its frequency has been gradually supplanted by the emergent Omicron sublineage NB.1.8.1, which the WHO designated as a ‘variant under monitoring’ on May 23, 2025 (13). The numerous additional mutations in the Omicron spike protein are considered to enhance resistance to neutralizing antibody responses induced by prior infection or vaccination (7, 8, 14, 15). Spike-specific T cell immunity to the Omicron variant appears to result in partial reduction (16–18); however, several studies have reported that Omicron and its subvariants may acquire further immune evasion capacity compared to previous variants of concern (VOCs) through new genetic changes (19–21). Cytotoxic T lymphocytes (CTLs), are the major effectors of antiviral T cell reactivity, and are known to play a pivotal role in effective viral clearance and attenuation of COVID-19 disease severity (22–25). Therefore, it is important to evaluate and understand CTL escape mutations in currently circulating SARS-CoV-2 variants to effectively prepare for future viral evolution.

Anti-viral CTL responses are initiated by recognizing CD8^+^ T cell epitopes via human leukocyte antigen (HLA) class I alleles (26, 27). Hence, studies on CTL epitopes are restricted to HLA subtypes, and most aim to identify antigenic peptides with high binding affinity to the corresponding HLA class I molecules (28–30). Mutations derived from variants within these epitopes have the potential to impair HLA binding and reduce CTL activation. Single and multiple mutations in SARS-CoV-2 VOCs could alter epitope-HLA binding and subsequently cellular immunity (20, 30–33). However, experimental evidence remains insufficient, and additional laboratory data are required to elucidate CTL-mediated immune evasion in the rapidly shifting landscape of SARS-CoV-2 variants.

The prolonged circulation of COVID-19 has led most individuals to develop hybrid immunity through vaccination and natural infection. BA.1 and BA.2, which drove the first and most extensive Omicron wave of breakthrough infections, are considered critical variants that constitute the immunological basis of hybrid immunity during the ongoing transition to endemicity. Such substantial exposure to heterologous antigens enhanced CTL responses against subsequent Omicron subvariants by promoting the recognition of variant-specific epitopes (34, 35). Thus, SARS-CoV-2 variants can evade pre-existing cellular immunity through genetic mutations, but subsequent antigenic exposure, such as infection and vaccination, may reshape T cell responses to the mutated epitopes. This highlights the need to assess whether CTL-mediated immune evasion confirmed in vaccinated individuals persists following breakthrough infection.

In the present study, we investigated the potential of single and multiple mutations in SARS-CoV-2 variants to evade pre-existing CTL responses in COVID-19 vaccine recipients. We employed bioinformatic tools to predict HLA-A*24:02-restricted epitopes, one of the most frequent HLA class I alleles globally, particularly in Northeast Asian populations such as South Korea (22.7% in South Korea, 37.8% in Japan, and 14.4% in China) (28, 30, 36–38). The antigenic peptides with high HLA binding affinity were screened from the SARS-CoV-2 spike protein, and a novel HLA-A*24:02-restricted epitope, LYNSASFSTF (LYN, S368-377), was subsequently identified through experimental validation. The four Omicron-derived mutations present in LYN led to reduced cellular immunity in vaccinated individuals. In particular, the T376A mutation, first identified in the BA.2 variant, appears to be the major contributor to the evasion of LYN-specific immune responses. The impaired cellular immunity was partially restored after BA.1/BA.2 breakthrough infections, likely due to improved recognition of the mutated epitope. Our data indicate that LYN may play a dual immunological role, functioning either as an immune-evasive hotspot or a cross-reactive epitope, highlighting its significance in vaccine design and immune surveillance.

## 2 Materials and methods

### 2.1 Study participants

Peripheral blood mononuclear cell (PBMC) samples were obtained from 32 participants enrolled in nationwide multicenter prospective cohorts (**Supplementary Table S1**) (39, 40). Participants received three doses of either the BNT162b2 or mRNA-1273 vaccine according to homologous vaccination schedules (n = 16 per vaccine group). Breakthrough infections were confirmed between 3 and 5 months after the third dose, based on polymerase chain reaction (PCR) or serological analyses (**Supplementary Table S2**). All participants in this study were continuously monitored, and no infections were detected before the Omicron breakthrough period (39–42), confirming that the breakthrough cases analyzed here represented primary SARS-CoV-2 infections. HLA typing was performed on PBMC samples to identify major HLA alleles prevalent in the Korean population, and the samples were classified into HLA-A*24:02–positive or –negative (**Supplementary Table S3**).

### 2.2 MHC-I binding affinity prediction

Before conducting the epitope prediction analysis, we reviewed previous studies on the expression frequency of HLA alleles in Koreans (37, 43, 44). Based on this review, we identified the HLA-A*24:02 allele as the most frequently expressed allele in the Korean population and conducted a predictive analysis using this allele as the primary focus. To predict the binding capacity of wild-type (WT) and mutant peptides to HLA molecules, the binding affinity between WT/mutant peptides and the HLA-A*24:02 allele was analyzed using the NetMHCpan 4.1 tool (45). We assessed the binding capacity of each 8–14-mer peptide from the SARS-CoV-2 ancestral spike protein (Accession No. YP_009724390.1) to HLA molecules, where strong binders were identified based on an EL-percentile rank threshold of 0.5 (N = 8,841) (30). The percentile rank of SARS-CoV-2 VOCs were based on the following GISAID sequences: Alpha (B.1.1.7, EPI_ISL_581117), Beta (B.1.351, EPI_ISL_660190), Gamma (P.1, EPI_ISL_811149), Delta (B.1.617.2, EPI_ISL_1409773), Omicron BA.1 (EPI_ISL_6590782.2) and Omicron BA.2 (EPI_ISL_9845731). In this analysis, deletion or insertion mutations in the amino acid sequence were removed from the final results (**Supplementary Table S4**) because of varying peptide lengths. Among the eight final selected peptides, QLP and GYQ were excluded due to the inability to synthesize dextramers. Consequently, T cell immune responses were analyzed for the remaining four peptides: TQL, VYF, LYN and NYN.

### 2.3 PBMC isolation

Blood samples were collected in citrate phosphate tubes and processed within 2 h of collection. PBMCs were isolated by centrifugation at 1,500 rpm for 10 min, suspended in freezing media (fetal bovine serum [FBS] containing 10% dimethyl sulfoxide [DMSO]). Cells were frozen at −80°C and stored in liquid nitrogen until further use.

### 2.4 HLA typing

HLA typing was performed using a previously developed polymerase chain reaction by sequence-specific primer (PCR-SSP) method (46). In brief, sample DNA was extracted from one vial of PBMCs using a DNeasy Blood & Tissue kit (Qiagen, Hilden, Germany) according to the manufacturer’s instructions. This DNA was used as a template for PCR amplification of the HLA-A gene using specific primers (primer sequences and related information are presented in **Supplementary Table S5**). The recommended cycling conditions from the manufacturer was used (Bioneer Corporation, Daejeon, Republic of Korea) PCR products were checked by agarose gel electrophoresis.

### 2.5 T cell expansion and dextramer staining

PBMCs were thawed and pulsed with 25 μL/mL ImmunoCult™ Human CD3/CD28/CD2 T cell Activator (STEMCELL Technologies, Vancouver, BC, Canada) and cultured for 10 days, adding 20 IU IL-2 (Roche, Basel, Switzerland) in ImmunoCult™-XF T cell expansion medium (STEMCELL Technologies) on day 3, 5 and 7. The proliferated T cells were washed once with RPMI-1640 (Gibco, Waltham, MA, USA) with 10% FBS (Gibco), 1% pen/strep (Gibco) media, followed by an additional wash with Dulbecco’s Phosphate−Buffered Saline (DPBS), and moved in 5 mL round bottom polystyrene tubes at 1 × 10^6^ cells/mL. Cells were pelleted and resuspended with PBS, as well as the corresponding dextramer (Immudex, Copenhagen, Denmark) at a final concentration of 10 μg per mL and incubated at 4°C for 20 min before adding the PE-conjugated anti-human CD3 antibody (eBioscience, San Diego, CA, USA) and FITC-conjugated anti-human CD8 antibody (BioLegend, San Diego, CA, USA), and incubating for an additional 15 min at 4°C. Dead cells were stained with LIVE/DEAD™ Fixable Aqua Dead Cell Stain Kit (Invitrogen, Waltham, MA, USA) for 20 min at 4°C. The stained cells were pelleted and washed three times with PBS before flow cytometry analysis (Beckman Coulter, CytoFLEX LX). Frequencies of antigen-specific T cells were calculated as the percentage of dextramer-positive cells within the CD8^+^ T cell population, with background subtraction performed using the corresponding fluorescence minus one (FMO) control.

### 2.6 Cell stimulation

Thawed PBMCs were incubated in RPMI 1640, GlutaMAX™ Supplement medium (Gibco) supplemented with 10% FBS (Gibco), 1% pen/strep (Gibco) with DNase I solution (STEMCELL Technologies) at room temperature for 15 min, followed by resting for 18 h at 37°C and 5% CO_2_. Subsequently, 1 × 10^6^ PBMCs were stimulated with 1 µg/mL peptide pools of SARS-CoV-2 spike protein (GenScript, Piscataway, NJ, USA) or artificially synthesized peptides (ANYGEN, Gwangju, Republic of Korea) in a 96-well U-bottom plate. For induction of T cell immune response by wild type or omicron (BA.1 and BA.2) spike protein, peptide pools of Miltenyi Biotec (Bergisch Gladbach, Germany) were used at a concentration of 1 µg/mL in cell stimulation (product information is listed in **Supplementary Table S6**). The background negative control was incubated with DMSO only. Stimulation with 50 ng/mL phorbol 12-myristate 13-acetate (Sigma-Aldrich, Darmstadt, Germany) and 1 µg/mL ionomycin (Sigma-Aldrich) served as positive control to demonstrate activation of T cells. All samples were incubated for 18 h at 37°C and 5% CO_2_.

### 2.7 Flow cytometric analysis of epitope-reactive T cells

After stimulation, the cells were stained for phenotypic lymphocyte markers. Stimulated cells were transferred into 5 mL round-bottom tubes, then washed and stained with the LIVE/DEAD™ Fixable Aqua Dead Cell Stain Kit (Invitrogen) at 4°C for 20 min. Subsequently, the samples were stained with CD3-BV786 (BD Biosciences), CD14-BV510 (BD Biosciences), CD19-BV510 (BD Biosciences), CD8-FITC (BioLegend), CD69-PE (eBioscience), CD137-APC (BioLegend), and CD137-APC (BioLegend) at 4°C for 30 min. The fluorescent intensity of the stained cells was measured using a CytoFLEX LX flow cytometer (Beckman Coulter). Flow cytometry data were analyzed using FlowJo (BD Biosciences, version 10.8.1). Reactive T cells were defined as CD69^+^CD137^+^ T cells within the total CD8^+^ T cell populations. Antigen-specific responses were quantified as background-subtracted frequencies, calculated by subtracting the values obtained from matched unstimulated (DMSO) controls. Surface marker-expressing reactive T cell subsets were analyzed using Boolean combination gating. The stimulation index (SI) was calculated as the ratio of the frequency of AIM^+^ cells after SARS-CoV-2 peptide stimulation to that of AIM^+^ cells in the DMSO control.

### 2.8 Docking simulation of HLA-A*24:02-peptide complex

To assess the structural interaction between HLA-A*24:02 and LYN peptides, molecular docking simulations were performed using the GalaxyPepDock web server (47). The crystal structure of HLA-A*24:02 (PDB ID: 8YZZ) was obtained in PDB format from the RCSB Protein Data Bank (https://www.rcsb.org/), and the peptide sequences were provided in FASTA format. GalaxyPepDock generated 10 docking models through energy-based optimization, and the model with the highest interaction similarity score was selected for further analysis. Hydrogen bond interactions between the peptide and HLA binding groove residues were identified using UCSF ChimeraX version 1.10 (48). Key interacting residues and hydrogen bond networks at the peptide–MHC interface were visually inspected and annotated for structural interpretation.

### 2.9 Quantification and statistical analysis

Statistical analyses were performed using GraphPad Prism 8.2.1 software and used non-parametric tests for all comparisons. Comparisons between two groups were analyzed using paired t-tests or Wilcoxon matched-pairs signed rank tests, and comparisons among three or more groups were evaluated using one-way ANOVA followed by Dunnett’s multiple comparison test. All statistical analyses were performed as two-sided test, and *P* < 0.05 was considered statistically significant. Due to multiple testing, p-values were considered descriptive.

## 3 Results

### 3.1 Enhanced CTL escape by BA.2 spike mutations in HLA-A24 donors

**Figure 1:**
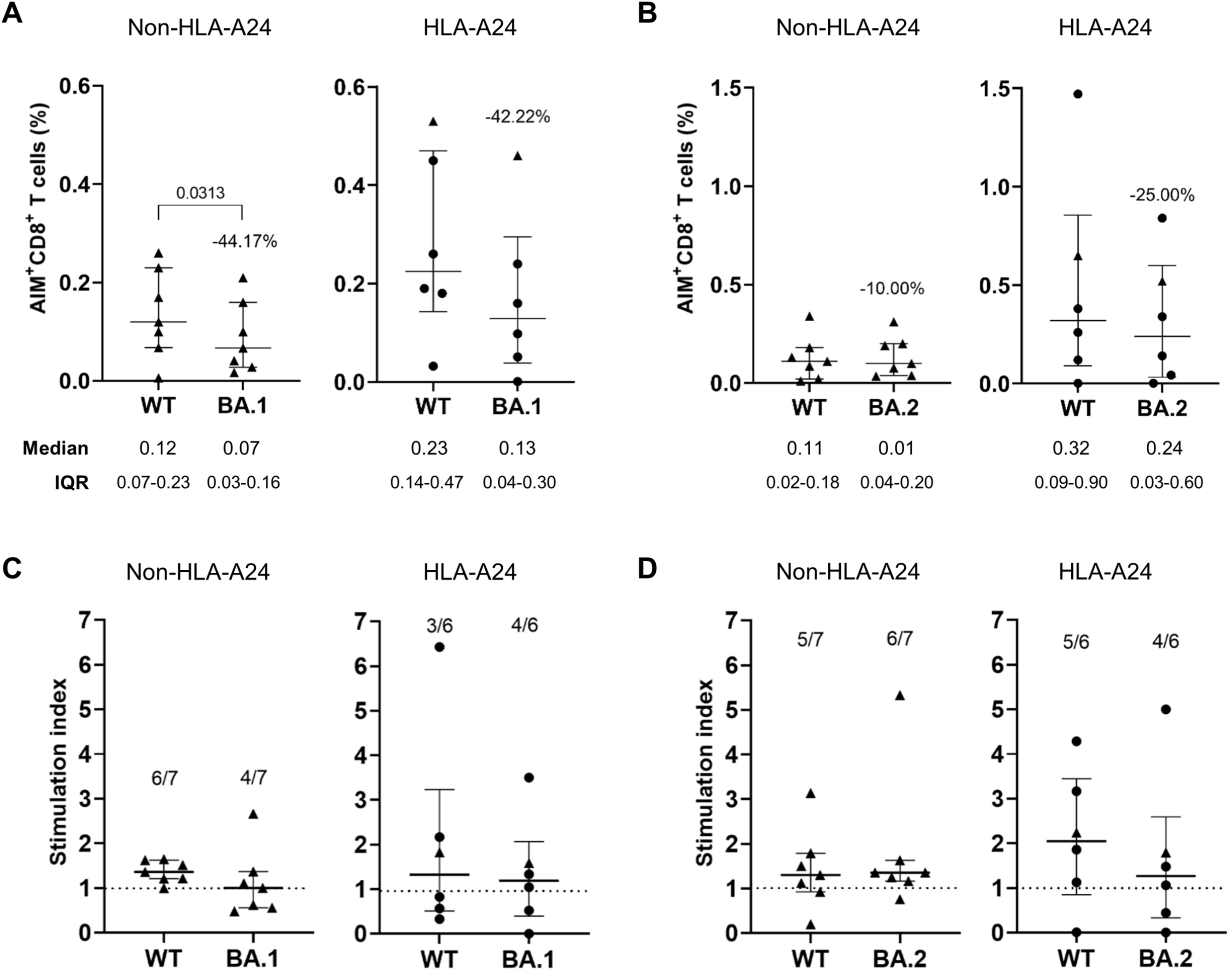
Augmented immune evasion from HLA-A*24:02-restricted CD8^+^ T cell responses by BA.2 mutations in COVID-19 vaccine recipients. **(A, B)** Spike-specific CD8^+^ T cell responses were measured by co-expression of the activation markers CD69 and CD137 using the activation-induced marker assays in BNT162b2 (n = 5, •) or mRNA-1273 (n = 8, ▴) vaccinated individuals with no history of SARS-CoV-2 infection. Peripheral blood mononuclear cells collected 3 months after the third dose were divided into HLA-A*24:02-positive (HLA-A24) and -negative (non-HLA-A24) groups, then stimulated with BA.1 **(A)** or BA.2 **(B)** mutated peptide pools and compared to responses induced by the corresponding wild-type (WT) peptides. The frequency of AIM^+^CD8^+^ T cells was background-subtracted using values from the unstained and DMSO-treated controls. Stimulation indices (SI) were calculated as the fold change in CD8^+^ T cell responses to BA.1 **(C)** or BA.2 **(D)** peptide pools relative to the frequency of activated T cells in the DMSO-treated negative control. Exact P values are shown in comparison with the reference peptide pools. **(A)**. The dotted lines in **(C, D)** represent a SI value of 1 as a cut-off point. All values are expressed as the median and the error bar indicates the interquartile range.

We assessed whether Omicron-derived spike mutations confer resistance to vaccine-induced CD8^+^ T cell reactivity using activation-induced marker (AIM) assays (**Figure 1**). PBMC samples were collected from participants 3 months after receiving three doses of either the BNT162b2 (Comirnaty, Pfizer/BioNTech) or the mRNA-1273 (Spikevax, Moderna) vaccine, HLA typed for major class I alleles prevalent in the Korean population, and subsequently classified into HLA-A*24:02-positive and -negative groups (**Supplementary Tables S1**, **S3**). To exclude the effect of non-mutated sequences, we used peptide pools covering only the mutated regions of the BA.1 or BA.2 spike protein, with corresponding ancestral sequences as reference controls. A representative gating strategy for the AIM assay are shown in **Supplementary Figure S1**. The reduction in CD8^+^ T cell responses to the BA.1 peptide pool had no significant difference between HLA-A*24:02 (–44.22%) and non-HLA-A*24:02 (–44.17%) donors (**Figure 1A**). In contrast, BA.2-derived mutations led to greater CTL escape in HLA-A*24:02 (–25.00%) donors than in non-HLA-A*24:02 (–10.00%) donors (**Figure 1B**). Consistent with this observation, the proportion of responders with SI > 1 (relative to the DMSO control) was lower in HLA-A*24:02-positive donors than in negative donors against BA.2-derived peptides (**Figures 1C, D**). Overall, these results suggest that spike-specific T cell immunity in HLA-A*24:02-positive vaccine recipients may be more affected by mutations derived from BA.2 than BA.1.

### 3.2 Identification of potential HLA-A24-restricted T cell epitopes harboring VOC spike mutations

**Figure 2:**
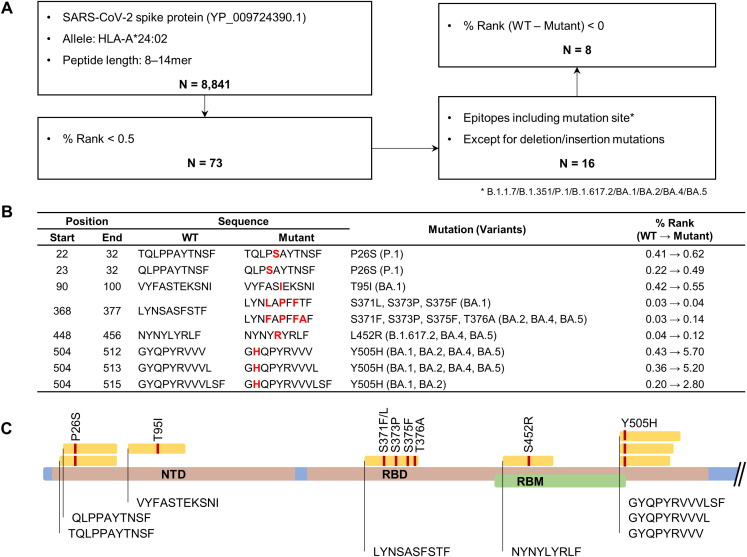
Bioinformatic prediction of HLA-A*24:02-restricted spike epitopes containing variant-associated mutations. **(A)** Flowchart for HLA-A*24:02-restricted epitope prediction. The epitope candidates in the ancestral SARS-CoV-2 spike protein (accession number YP_009724390.1) were selected based on the binding affinity score (percentile rank value, % rank) to the HLA-A*24:02 allele using the NetMHCpan EL 4.1 tool. **(B)** Original and mutant sequences of final epitope candidates. Amino acid changes are marked in bold red. **(C)** Schematic diagram showing the locations of the predicted epitopes and their mutation sites in the spike protein.

To determine which mutations contribute to HLA-A*24:02-mediated cellular immune evasion in vaccinated individuals, we predicted potential T cell epitopes for the spike protein of ancestral SARS-CoV-2 (WT) and examined whether they harbor VOC mutations. Using the NetMHCpan 4.1 EL tool (45), a total of 8,841 peptides, ranging from 8 to 14 amino acids in length and likely to bind to HLA-A*24:02, were selected from the WT spike protein (**Figure 2A**). Then, we screened 73 potential epitopes with high predicted affinity to HLA-A*24:02 (% Rank < 0.5), of which 16 candidates contain VOC mutations (**Supplementary Table S4**). The final putative epitopes were selected based on the decreased binding affinity (increased % Rank) in the mutant peptides compared to the original peptides. This multi-step filtering ensured that only peptides predicted to exhibit reduced HLA-A*24:02 binding due to variant-specific mutations were selected for experimental validation (**Figure 2B**; **Supplementary Table S4**). Previous studies have consistently confirmed the peptide NYNYLYRLF (NYN) as an HLA-A*24:02-restricted epitope in vaccinated and convalescent individuals (31, 33, 49, 50), whereas the other epitopes have been less studied and require further validation. Of note, LYNSASFSTF (LYN) was predicted to have strong binding affinity to HLA-A*24:02, similar to NYN (% Rank, LYN 0.03 and NYN 0.04), and the affinity score of LYN (0.03) was more affected by mutations derived from BA.2 (0.12) than BA.1 (0.04). Among the eight final epitope candidates, four peptides, including LYN and NYN, were located in the receptor binding domain (RBD), and the other three peptides, such as TQLPPAYTNSF (TQL) and VYFASTEKSNI (VYF), were found on the N-terminal domain (**Figure 2C**).

### 3.3 Validation of the HLA-A24-restricted epitope LYN in vaccinated individuals

**Figure 3:**
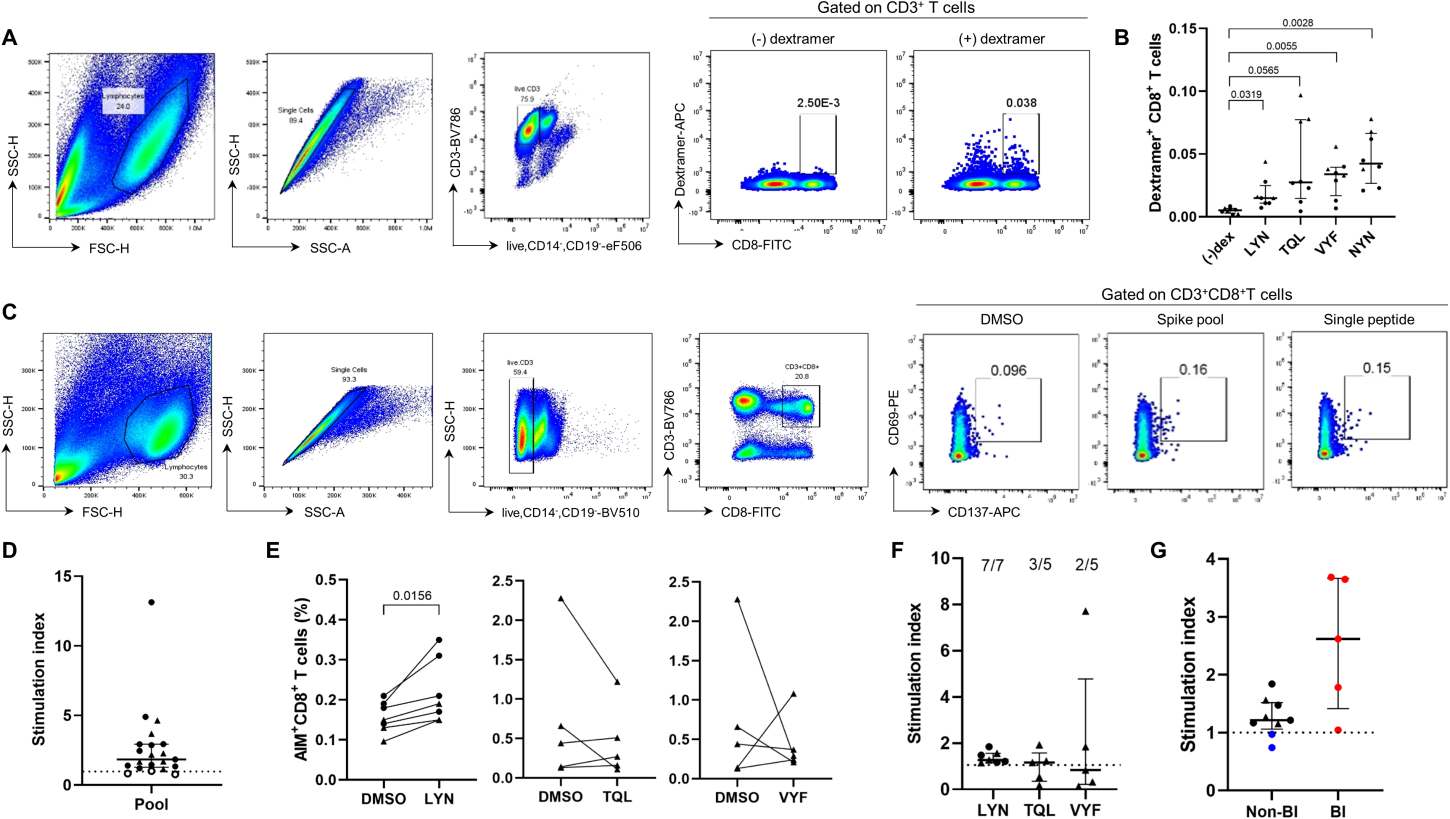
Experimental validation of the HLA-A24-rstricted epitope inducing CTL responses in COVID-19 vaccine recipients. **(A, B)** Validation of epitope candidates by peptide-MHC dextramer staining in the T cell populations expanded from HLA-A*24:02-genotyped peripheral blood mononuclear cells (PBMCs). The frequency of peptide-specific dextramer^+^CD8^+^ T cells was measured in BNT162b2 (n = 2, •) or mRNA-1273 (n = 6, ▴) vaccine recipients. **(A)** is the representative flow cytometry plots for **(B)** (–)dex represents an FMO control in which all antibodies were included except for dextramer–APC. **(C–F)** The peptides LYN, TQL, and VYF were further examined by AIM assay in BNT162b2 (•) or mRNA-1273 (▴) vaccinated individuals. HLA-A*24:02-positive PBMCs were stimulated with wild-type peptide pool or the epitope candidate peptides, and the percentages of antigen-specific CD8^+^ T cells were analysed using CD69 and CD137 markers. **(C)** is the representative flow cytometry plots for **(D, E)**. Stimulation indices calculated as fold change in activated T cell frequencies by each peptide relative to the dimethyl sulfoxide (DMSO)-treated negative control are presented **(F)**. **(G)** Stimulation indices (SI) based on LYN-specific CD8^+^ T cell frequency were compared between HLA-matched (black) and –mismatched (blue) PBMC samples using AIM assay. Additionally, HLA-A*24:02-positive samples from vaccinated individuals who experience breakthrough infection (BI, red) were analysed. Exact P values are shown in comparison with the fluorescence minus one (FMO) control lacking dextramer-APC **(B)** or with DMSO-treated samples **(E)**. SI were calculated as the fold change in activated T cell frequencies in response to each peptide relative to the DMSO-treated negative control. The dotted lines in **(D, F, G)** represent a SI value of 1 as a cut-off point. All values are expressed as the median and the error bar indicates the interquartile range in **(D, F, G)**.

We attempted to generate peptide-MHC dextramer complexes to examine whether the predicted epitope candidates could elicit specific CD8^+^ T cell responses in PBMCs from HLA-matched vaccinated individuals. Among the eight candidate peptides (**Figure 2B**), half were successfully produced (TQLPPAYTNSF, VYFASTEKSNI, LYNSASFSTF, and NYNYLYRLF), while the failed ones were excluded from subsequent experiments (QLPPAYTNSF, GYQPYRVVV, GYQPYRVVVL, and GYQPYRVVVLSF). HLA-A*24:02-positive PBMCs from COVID-19 mRNA vaccine recipients were cultured with T cell activators (CD3/CD28/CD2) and IL-2 for 10 days to expand T cell populations (**Supplementary Table S3**). The expanded T cells were stained with peptide-MHC dextramers, and the distribution of dextramer-positive CD8^+^ T cells was analyzed using flow cytometry (**Figure 3A**). The percentage of CD8^+^ T cells stained by the NYN-MHC dextramer, as a positive control, was significantly increased compared with the dextramer-unstained control (**Figure 3B**, *P* = 0.0028). LYN, TQL and VYF also substantially elevated the percentages of dextramer-positive T cells for each peptide (*P* = 0.0319, 0.0565 and 0.0055, respectively).

The newly identified epitope candidates (LYN, TQL, and VYF) were further validated using AIM assays (**Figure 3C**). NYN, which had already been verified as a HLA-A*24:02-restricted epitope (31, 33, 49, 50), was excluded from this experiment. To accurately analyze the potential epitope peptide-specific T cell responses, HLA-A*24:02-positive PBMCs that showed sufficient T cell reactivity to the ancestral strain-based peptide-pool were selected (filled circles and triangles in **Figure 3D**), and the frequency of antigen-specific CD8^+^ T cells was determined by co-expression of CD69 and CD137 after stimulation with the candidate peptides (**Figure 3E**). The peptide LYN significantly elevated CD8^+^ T cell responses from vaccinated individuals (*P* = 0.0156). All samples tested in the experiment consistently activated cellular immunity in response to LYN, although the T cell reactivity was relatively lower compared to other candidates. In contrast, the TQL and VYF peptides failed to increase CD8^+^ T cell responses in most PBMC samples (both *P* = 0.8125), which was consistent with the SI results in **Figure 3F**. Breakthrough SARS-CoV-2 infections (BI) after the third vaccine dose (red circles) enhanced LYN-specific T cell responses, whereas HLA-mismatched donors (blue circles) did not respond to the LYN peptide (**Figure 3G**). Collectively, these findings support that the peptide LYN is an immunodominant epitope frequently activated in COVID-19 vaccine recipients.

We attempted to experimentally validate three predicted epitope candidates, but unlike LYN, TQL and VYF could not be confirmed as HLA-A*24:02-restricted epitopes. The binding scores of TQL and VYF narrowly met the cut-off criterion (< 0.5), with scores of 0.41 and 0.42, respectively. In contrast, LYN exhibited a markedly lower score of 0.03, which was comparable to that of the previously validated epitope NYN (0.04). Although additional data are required to establish accurate criteria, our results suggest that the % Rank cut-off of < 0.5 may lack sufficient stringency and could be refined to a lower value, such as < 0.4, for screening HLA-A*24:02 epitopes using bioinformatics tools.

### 3.4 Reduction of LYN-specific CTL responses by the S375F and T376A mutations

**Figure 4:**
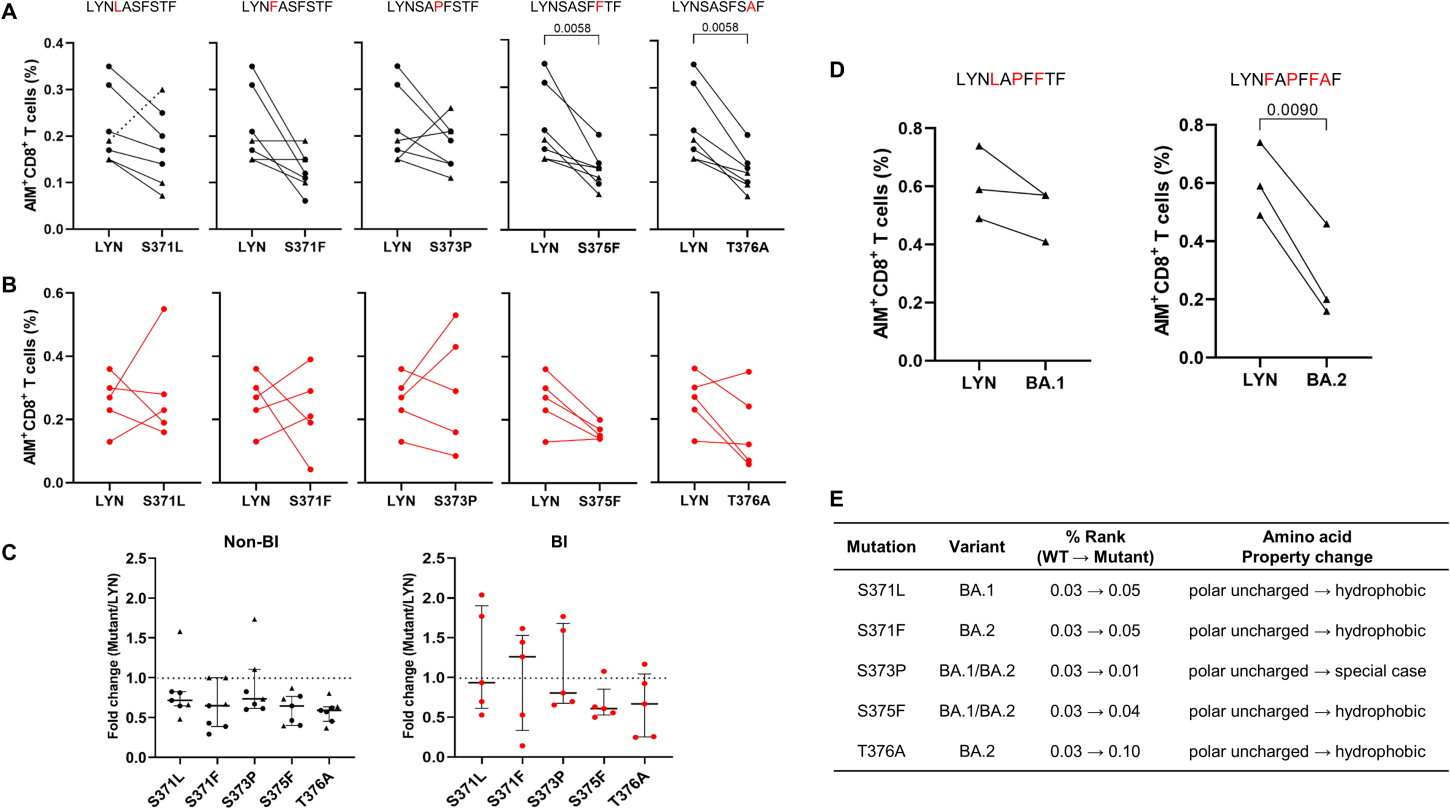
T cell immune evasion induced by single or combined mutations within the LYN epitope. **(A–C)** According to breakthrough infection (BI) history following a third BNT162b2 (•) or mRNA-1273 (▴) vaccine dose, HLA-A*24:02-positive peripheral blood mononuclear cells (PBMCs) were classified into non-BI **(A)** and BI **(B)** groups. The PBMC samples collected 5–8 months after the third vaccination were stimulated with LYN or its mutated peptides, and the frequency of activated T cells in response to each peptide was analysed using the AIM assay. The fold change in the CD8^+^ T cell responses induced by each mutant peptide relative to the wild-type peptide (LYN) is presented in **(C)**. **(D)** HLA-A*24:02-genotyped PBMCs in a non-BI group (5 months after the third mRNA-1273 vaccination, n = 3, ▴) were stimulated with LYN or its multi-mutation peptides, and then activated CD8^+^ T cells responding to each peptide were measured using the AIM assay. These mutant peptides harbour multiple mutations derived from Omicron BA.1 and BA.2, respectively. **(E)** Changes in the binding affinity score (percentile rank value, % rank) to HLA-A*24:02 allele caused by each single mutation on LYN were predicted using the NetMHCpan 4.1 EL tool with the corresponding amino acid properties. Exact P values are shown in comparison with the wild-type LYN peptide **(A, D)**. Statistical differences were determined using the Wilcoxon matched-pairs signed rank test for **(A)** and paired t test for **(D)**. Amino acid substitutions in the LYN peptide of BA.1 and BA.2 are highlighted in red **(A, D)**, and the dotted lines in **(C)** represent a fold-change value of 1 as a cut-off point. All values are expressed as the median and the error bar indicates the interquartile range in **(C)**. Non-specific binding in the AIM responses was excluded by gating based on the isotype control and DMSO-treated negative control.

The HLA-A*24:02-restricted epitope LYN, identified through dextramer staining and AIM assay (**Figures 3B, E**), harbors multiple mutations derived from Omicron variants (**Figure 2C**). To experimentally determine whether the variant-associated mutations can diminish vaccine-induced cellular immunity, we compared the frequencies of activated CD8^+^ T cells after stimulation with WT or mutant peptides, each containing a single mutation derived from BA.1 or BA.2, using an AIM assay (see **Supplementary Figure S1** for A representative gating strategy). In HLA-A*24:02-matched PBMCs collected from vaccinated recipients, the S375F (BA.1/BA.2) and T376A (BA.2) mutations resulted in a significant reduction (both *P* = 0.0313) in the percentage of peptide-specific CD8^+^ T cells (**Figure 4A**). The mutant peptides, containing S371L/F or S373P, could confer resistance to cellular immunity in five or six out of the seven vaccinated individuals (S371L: *P* = 0.0313, excluding one outlier indicated by a dotted line in **Figure 4A**). Notably, applying a multiple-comparison framework to the same data (**Supplementary Figure S2**) produced higher statistical significance for T376A (*P* = 0.0092) versus wild-type. In contrast, SARS-CoV-2 infection following the third vaccination led to inconsistent responses between WT and mutant peptides (**Figure 4B**). Fold changes were also calculated in BI and non-BI samples relative to the WT peptide, as shown in **Figure 4C**. We evaluated the combinatorial effects of immune evasion by naturally occurring mutations in the LYN of BA.1 and BA.2 (**Figure 4D**). The combination of S371F, S373P, S375F, and T376A (BA.2 mutant) resulted in a substantial reduction (*P* = 0.0090) in the frequency of LYN-specific T cell responses, while the combinatorial effect of S371L, S373P, and S375F (BA.1 mutant) was not statistically significant (*P* = 0.1750). Consistent with the AIM assay results, bioinformatic analysis predicted that these single (**Figure 4E**) and multiple (**Figure 2B**) amino acid substitutions could reduce the binding affinity between the peptide LYN and HLA-A*24:02. All single mutations except S373P increased the % Rank compared to WT, and the combination of multiple substitutions in BA.2 was predicted to show weaker binding affinity to HLA-A*24:02 than that of BA.1. Notably, the T376A mutation is the only substitution in BA.2 that sets it apart from BA.1, since S371L and S371F share identical % Rank values (0.05). Considering that the affinity score of LYN was most affected by the T376A mutation (from 0.03 to 0.10), this amino acid change may be a crucial factor for immune evasion from HLA-A*24:02-mediated cellular immunity.

**Figure 5:**
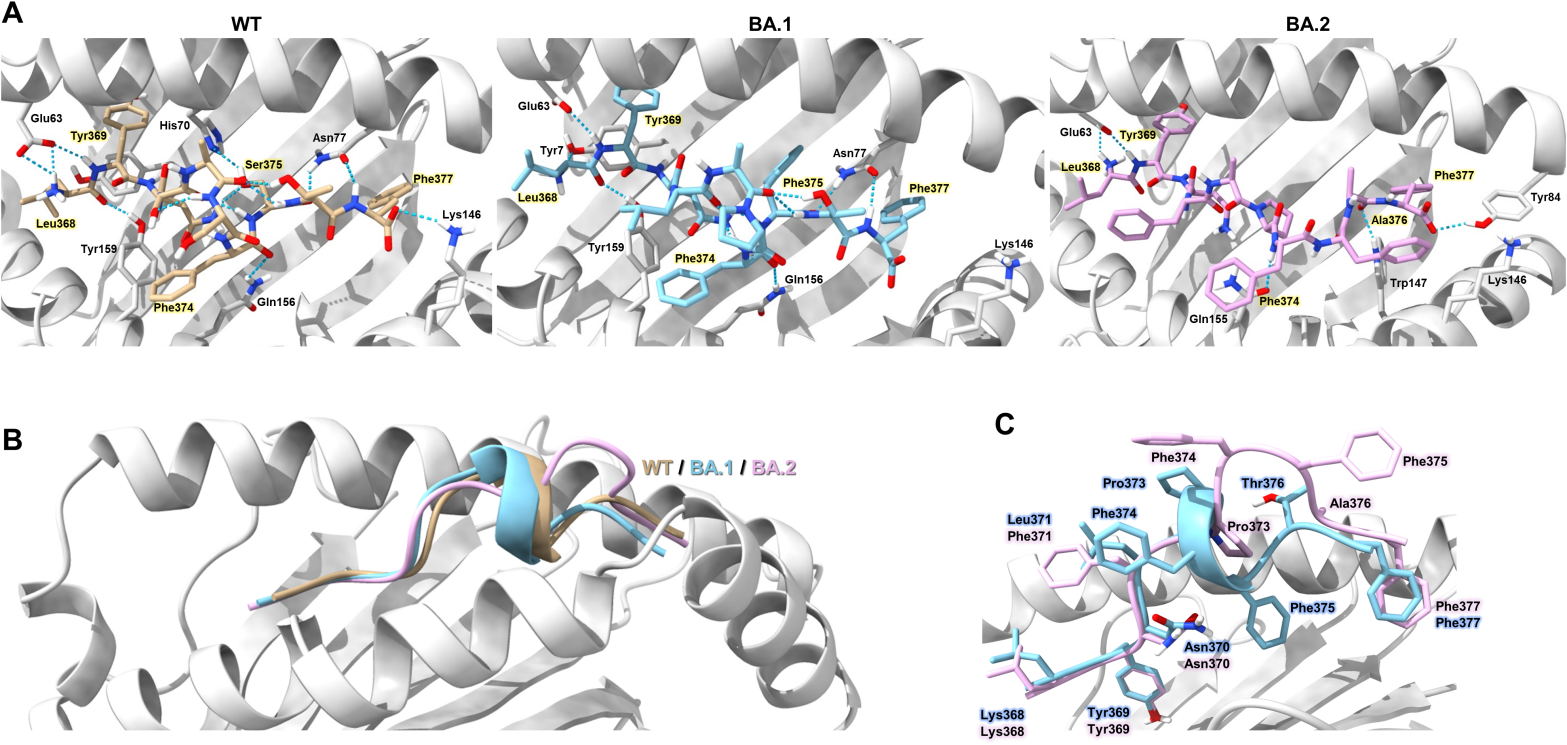
Structural model of HLA-A*24:02-LYN peptide complex based on docking simulation. **(A)** Overview of the structure model showing hydrogen bond interactions (blue dotted lines) between HLA-A*24:02 and the LYN epitope or its mutants. Individual peptides are coloured light brown (WT), sky blue (BA.1), and pink (BA.2). Hydrogen bond-forming residues of the HLA molecule that interact with peptide residues are annotated in the figure. Key peptide residues forming direct hydrogen bonds with the HLA binding groove are highlighted in yellow. **(B)** Structural differences among the WT and mutant peptides are illustrated as cartoon representations, with each peptide coloured according to the scheme in **(A)**. **(C)** Comparison of C-terminal conformations in BA.1 and BA.2 peptides, with each residue colored sky blue (BA.1) or pink (BA.2).

To elucidate the molecular mechanism of immune evasion by Omicron-derived mutations in the LYN epitope, we analyzed the structural interactions between HLA-A*24:02 and LYN peptides using the top-ranked GalaxyPepDock model (**Figure 5A**). Structural analysis revealed that the WT LYN peptide forms eight strong hydrogen bond pairs with the HLA-A*24:02 binding groove, whereas the BA.1-derived mutant peptide exhibits the loss of two hydrogen bonds involving Glu63–P1 and Lys146–P10 within the major anchor pockets (51). Further mutations in BA.2 resulted in the loss of an additional hydrogen bond and induced more pronounced conformational changes at the peptide C-terminus than those observed in BA.1 (**Figures 5B, C**). These results suggest that Omicron-derived mutations in the LYN epitope compromise the binding affinity of the HLA-A*24:02–LYN complex by disrupting hydrogen bonds and inducing structural alterations.

## 4 Discussion

In this study, we analyzed the T cell immune escape characteristics of SARS-CoV-2 variants to elucidate their effects on pre-existing CTL responses in COVID-19 vaccinated individuals. The HLA-A*24-restricted epitope LYN, located in the spike RBD, was identified and characterized as having four mutations derived from BA.1 and BA.2. We demonstrated that the newly emerged BA.2 mutation T376A can further reduce LYN-specific T cell responses compared to BA.1. Therefore, our findings suggest that Omicron variants may accumulate genetic mutations within the same CTL epitopes during their evolutionary process, potentially leading to additional loss in pre-existing cellular immunity.

The HLA-A*24:02 allele is one of the most common HLA class I subtypes globally, and particularly in East Asian populations (28, 36). Thus, it offers broad population coverage in studies of CTL-mediated immune responses. Interestingly, this study presented that cellular immune evasion induced by BA.2 spike mutations was more pronounced in HLA-A*24:02 donors (–20.0%) than in non-HLA-A24 donors (–10.0%). A similar trend for BA.2.86 variant was observed in HLA-A*24:02 convalescents, according to a recent study on T cell epitope hotspots (20). The observation that dominant SARS-CoV-2 variants exhibit enhanced cellular immune evasion in HLA-A*24:02, a globally predominant HLA allele, may not be a mere coincidence. Collectively, these findings highlight the need for close monitoring of HLA-A*24:02-restricted epitopes and their susceptibility to immune escape by newly emerging variants.

A prior study predicted LYN as an HLA-A24:02-restricted epitope through in silico analysis and experimentally validated it in healthy donors using a dendritic cell–peptide–PBMC co-culture system (52). In contrast, a separate investigation in convalescent COVID-19 patients naturally exposed to SARS-CoV-2 found no activation of LYN-specific cellular immunity, suggesting that this epitope may not be elicited by infection (53). Our data reveal robust LYN-specific CD8^+^ T cell responses in HLA-A24:02-positive vaccine recipients, establishing its role as a CTL epitope in humans. As most individuals during the COVID-19 pandemic experienced their primary antigen exposure through vaccination rather than infection, and given the high global prevalence of HLA-A*24:02, it is likely that a substantial proportion of the world’s population harbors LYN-specific immune responses.

The LYN sequence was conserved in earlier VOCs, but three amino acid substitutions—S371L, S373P, and S375F—were observed in the BA.1 variant (54). In the subsequent BA.2 variant, serine at position 371 was replaced by phenylalanine (S371F), and an additional mutation occurred at position 376 (T376A). These Omicron substitutions, positioned in the RBD loop targeted by class 4 antibodies (55–57), have prompted focused research into their role in antibody escape (54, 58). However, our findings suggest that genetic mutations at key neutralizing antibody binding sites could partially contribute to cellular immune evasion and might have been selectively retained during viral evolution, alongside other immune-driven pressures such as antibody responses. Evaluation of the BA.1-derived substitutions confirmed that only S375F significantly reduced LYN-specific T cell responses, while the other two induced minor decreases. Among BA.2-derived mutations, S371F induced a reduction in T cell responses comparable to that of S371L in BA.1, whereas the newly acquired T376A caused a substantial degree of immune evasion. Furthermore, the combined effects of multiple substitutions revealed that BA.2 led to a greater impairment of LYN-specific CTL immunity than BA.1, supporting the role of BA.2-specific T376A in enhancing immune escape. This pattern also applied to the HLA-A*24:02-restricted epitope NYN, which exhibited reduced T cell responses due to the BA.2.86-derived mutations (N450D and L452W), and the additional L455S substitution in the JN.1 variant further enhanced immune evasion (20). While the stepwise accumulation of mutations in both LYN and NYN resulted in reduced recognition by HLA-A*24:02–restricted T cells, this does not imply a complete loss of protection, as T cell–mediated immunity typically involves multiple epitopes that contribute collectively to host defense. Nevertheless, the identification of such T cell epitope hotspots highlights their potential value as indicators for monitoring how SARS-CoV-2 evolves to evade pre-existing cellular immunity.

After completing a three-dose COVID-19 vaccination, participants in the BI group experienced SARS-CoV-2 infection during the Omicron BA.1 and BA.2 waves. We confirmed that the Omicron infection increased LYN-specific T cell responses and mitigated the immune evasion caused by epitope mutations. Previous studies also showed that exposure to different variants enhanced the magnitude and breadth of cellular immunity in vaccinated individuals (34, 35). They suggested that vaccine breakthrough infection can induce CTL responses to new variant-specific epitopes, potentially cross-reactive with other strains sharing the same mutations. Moreover, exposure to SARS-CoV-2 variants has been reported to reshape immunity across CD4^+^ T cells, antibody responses, and natural killer cells (40, 59, 60). The LYN epitope acquired three mutations (S371L, S373P, and S375F) in early Omicron BA.1, which were further altered in BA.2 by a replacement with S371F and the addition of T376A (54). Since BA.2, the mutational profile of the LYN epitope has remained largely conserved up to the currently dominant LP.8.1 variant, except for a transient L361I substitution observed in XBB lineages (7, 61–63). Taken together, our findings suggest that LYN-specific immune responses were diminished by the emergence of Omicron BA.1/BA.2, but may have been subsequently restored and maintained through variant-adapted vaccination or breakthrough infection, given the conserved mutational profile observed since BA.2. Nevertheless, the LYN epitope remains at risk of future immune escape, as demonstrated by the additional impairment of CTL responses to the NYN epitope caused by JN.1-derived mutations.

As COVID-19 transitions into an endemic phase, this study underscores the importance of characterizing immunodominant CTL epitopes and immune escape mutations in major SARS-CoV-2 variants to inform long-term preparedness for future viral evolution. The newly identified HLA-A*24:02-restricted epitope LYN accumulated sequential mutations from BA.1 to BA.2, leading to enhanced evasion of cellular immunity. This site has remained largely conserved in the dominant variants, raising the possibility that T cell responses to the mutated LYN epitope may have been restored through repeated exposure to variant antigens via vaccination and infection. Further studies, however, are needed to validate this hypothesis, and additional CTL escape mutations may arise in future emerging variants. Overall, our findings suggest that the newly identified LYN epitope may represent a critical hotspot in SARS-CoV-2-specific cellular immunity, highlighting its potential relevance for future vaccine design and T cell-based immune monitoring.

South Korea maintained a low incidence of COVID-19 through high vaccination coverage and coordinated public health measures with strong community participation; however, the emergence of Omicron BA.1/BA.2 caused a surge of breakthrough infections. Our study population—comprising vaccine recipients who remained uninfected and those subsequently infected with Omicron—thus reflects the representative conditions of this period. For broad applicability, the analysis focused on HLA-A*24:02, one of the most prevalent HLA class I alleles in both the Korean population and worldwide. Regional epidemiological patterns differed, but the Omicron wave showed broadly comparable features worldwide, with widespread vaccination followed by large-scale breakthrough infections. Therefore, the HLA-A24:02-restricted epitopes and associated immune escape mutations identified in this study are not confined to the Korean context but provide broader insights into how current and emerging SARS-CoV-2 variants evade immunity and inform preparedness strategies, remaining highly relevant in the ongoing endemic phase where the virus continues to circulate and evolve.

This study was limited by the number of available samples; only PBMCs collected up to 5 months post-vaccination or breakthrough infection were included in the analysis. Although vaccine effectiveness may decrease over time following vaccination, cellular immunity against SARS-CoV-2 is reported to persist long-term (64, 65), and the samples used in this study showed consistent results in ELISpot analysis. The cohort size was relatively small (n = 32), and the sex distribution was uneven, with all participants in the mRNA-1273 group being female, which may limit the generalizability of the findings. Nevertheless, future studies with larger and more balanced cohorts will be valuable for validating and extending these findings. Our study focused on HLA-A*24:02, a widely distributed allele with broad population coverage, but it does not represent the full diversity of HLA class I types. To complement this, follow-up studies are being conducted for other prevalent alleles, such as HLA-A*02:01, using a similar experimental framework to that employed for HLA-A*24:02. Because computational screening relies on predicted binding affinity thresholds, it is possible that functionally relevant HLA-A*24:02–restricted epitopes were not identified in this study. In addition, while the efficient delivery and presentation of synthetic peptides pose practical challenges for functional validation, we partially addressed this limitation by incorporating dextramer staining as a complementary approach. This study suggests that BA.1/BA.2 breakthrough infections may contribute to improved recognition of mutated LYN epitopes, highlighting the need for mechanistic investigations, including TCR repertoire sequencing. SARS-CoV-2 infections were confirmed in study participants by PCR or serologic analysis (40); however, the infecting viral strains could not be identified due to a lack of sequencing data. Omicron BA.1 and BA.2 were considered the most likely infecting variants based on the breakthrough infection period and dominant circulating strains. Nevertheless, infection with other variants cannot be ruled out.

## Data Availability

The raw data supporting the conclusions of this article will be made available by the authors, without undue reservation.
